# High γ Activity in Cortex and Hippocampus Is Correlated with Autonomic Tone during Sleep

**DOI:** 10.1523/ENEURO.0194-21.2021

**Published:** 2021-11-17

**Authors:** Abdulwahab Alasfour, Xi Jiang, Jorge Gonzalez-Martinez, Vikash Gilja, Eric Halgren

**Affiliations:** 1 Department of Electrical Engineering, College of Engineering and Petroleum, Kuwait University, Kuwait City, Kuwait 13060; 2 Department of Electrical and Computer Engineering, University of California at San Diego, La Jolla, CA 92093; 3 Department of Neurosciences, University of California at San Diego, La Jolla, CA 92093; 4 Department of Neurological Surgery and Epilepsy Center, University of Pittsburgh, Pittsburgh, PA 15260; 5 Department of Neurosciences, Department of Radiology, University of California at San Diego, La Jolla, CA 92093

**Keywords:** autonomic nervous system, electrophysiology, heart rate variability, high γ, human cortex, sleep

## Abstract

Studies in animals have demonstrated a strong relationship between cortical and hippocampal activity, and autonomic tone. However, the extent, distribution, and nature of this relationship have not been investigated with intracranial recordings in humans during sleep. Cortical and hippocampal population neuronal firing was estimated from high γ band activity (HG) from 70 to 110 Hz in local field potentials (LFPs) recorded from 15 subjects (nine females) during nonrapid eye movement (NREM) sleep. Autonomic tone was estimated from heart rate variability (HRV). HG and HRV were significantly correlated in the hippocampus and multiple cortical sites in NREM stages N1–N3. The average correlation between HG and HRV could be positive or negative across patients given anatomic location and sleep stage and was most profound in lateral temporal lobe in N3, suggestive of greater cortical activity associated with sympathetic tone. Patient-wide correlation was related to δ band activity (1–4 Hz), which is known to be correlated with high γ activity during sleep. The percentage of statistically correlated channels was weaker in N1 and N2 as compared with N3, and was strongest in regions that have previously been associated with autonomic processes, such as anterior hippocampus and insula. The anatomic distribution of HRV-HG correlations during sleep did not reproduce those usually observed with positron emission tomography (PET) or functional magnetic resonance imaging (fMRI) during waking. This study aims to characterize the relationship between autonomic tone and neuronal firing rate during sleep and further studies are needed to investigate finer temporal resolutions, denser coverages, and different frequency bands in both waking and sleep.

## Significance Statement

Studies in animals have shown that the autonomic nervous system (ANS) sets the operating mode of all the organ systems in the body, including the central nervous system. We show here that high γ band activity (HG) in widespread cortical and hippocampal regions is correlated with heart rate variability (HRV) in humans during sleep. The correlation was especially profound in sites that have previously been associated with autonomic and emotional regulation. The direction of change varied between forebrain locations, indicating the existence of sympathetic and parasympathetic modulating structures. The percentage of correlated channels between autonomic tone and cortical activity was greatest in the deepest stages of slow-wave sleep. Overall, this study characterizes in humans a foundational link in the unity of mind and body.

## Introduction

The general state of the human organism is regulated by the autonomic nervous system (ANS) through afferent and efferent pathways. These pathways modulate various functions in the human body, such as digestion, blood pressure, heart rate, urination, sexual arousal, and others. A healthy ANS is key to maintain the balance of all these functions, adapt to different environmental stimuli, and keep the body in homeostasis. Two main divisions ensure this, the sympathetic and parasympathetic divisions. The sympathetic system is responsible for priming our bodies for a fight or flight response. In contrast, the parasympathetic system promotes energy conservation and digestion. These systems are not only influencing the waking brain but are responsible for a healthy and regenerative sleep cycle (Castro-Diehl et al., 2016; Tobaldini et al., 2017; Zoccoli and Amici, 2020).

Within the brain, ascending pathways modulate the level of cortical activity, notably via noradrenergic fibers from the locus coeruleus and cholinergic fibers from the nucleus basalis (Samuels and Szabadi, 2008; Mesulam, 2013). These nuclei are functionally connected with the ANS, which may treat the cerebral cortex as another internal organ to be maintained at an optimal level (Samuels and Szabadi, 2008; Mesulam, 2013). In mice and monkey, pupillary dilation, which could be used as an index of autonomic modulation, was shown to be correlated with the firing of noradrenergic and cholinergic neurons; phasic pupillary dilations track the firing of noradrenergic axons, whereas sustained dilations track the firing of cholinergic axons (Joshi et al., 2016; Reimer et al., 2016).

In humans, multiple studies have established a link between the central and ANSs, mainly by examining correlations in task-induced positron emission tomography (PET) and functional magnetic resonance imaging (fMRI) responses and autonomic measurements. Meta-analyses (Thayer et al., 2012; Beissner et al., 2013; Macey et al., 2016; Sklerov et al., 2019) showed that the blood oxygenation level-dependent (BOLD) response in multiple cortical, limbic and hippocampal structures as well as the default mode network (DMN) are correlated with heart rate variability (HRV) when observed through fMRI task-based and task-free experimental paradigms. However, the origin of this correlation is unclear. It could be a modulation of cortical firing by ascending noradrenergic and/or cholinergic pathways associated with the autonomic system, modulation of brainstem autonomic structures by corticofugal influences, or a viscerosensory response by cortical neurons. Alternatively, the cortical BOLD modulation may not be because of neural activity, but rather to direct effects of the ANS on blood flow mediated by its well-known effects on blood pressure, heart rate, and vasodilation (Özbay et al., 2019). This potential confound is mitigated in electrophysiological studies. A recent study found that the firing rate of human cingulate and parahippocampal gyrus neurons may show a negative or positive correlation with the heart rate (Kim et al., 2019). There have also been studies that investigate the CNS-ANS relationship using electroencephalography (EEG) recordings throughout sleep (de Zambotti et al., 2016, 2018). However, because of the limited temporal resolution of fMRI studies, the possibility that the BOLD signal can be influenced by local neurovascular modulation, and the lack of spatial resolution with EEG-sleep studies, little is known about the relationship between the ANS and CNS during sleep.

In this study, we aimed to investigate the correlation of HRV, which is used as a metric for the autonomic tone, with intracranial recordings of cortical and hippocampal activity as indexed by high γ band activity (HG), which is known to be positively correlated with neuronal firing rate (Mukamel et al., 2005; Manning et al., 2009; Ray and Maunsell, 2011; Miller et al., 2014), during sleep. We focused on sleep because it is relatively free of other activities (such as eating, talking, or moving) which could influence either forebrain activity, autonomic state, or both. Additionally, we investigated the effect of the sleep stage on the correlation between autonomic tone and neural activity in different anatomic regions, since sleep stage and autonomic control are known to be correlated (Trinder et al., 2001; Monti et al., 2002). We also investigate the effects of δ band activity on the correlation between autonomic tone and HG because of the fact that δ band up states and down states (DSs) modulate HG in humans during sleep (Csercsa et al., 2010; Halgren et al., 2018).To our knowledge, this is the first attempt to investigate the connection between the ANS and CNS during sleep using intracranial EEG recordings.

## Materials and Methods

### Patient selection

Sixteen patients with long-standing drug-resistant partial seizures underwent stereo EEG (sEEG) depth electrode implantation to localize seizure onset and direct surgical treatment. We selected the 16 patients (10 female) from a group of 54 for this study that displayed minimal hippocampal pathology and had electrocardiogram (ECG) recordings. The average age was 30 and ranged from 16 to 58 years old. sEEG implantation was based entirely on clinical needs (Gonzalez-Martinez et al., 2013). All patients gave fully informed consent for data usage as monitored by the local Institutional Review Board, following clinical guidelines and regulations at Cleveland Clinic.

### Electrode localization

**Figure 1.**
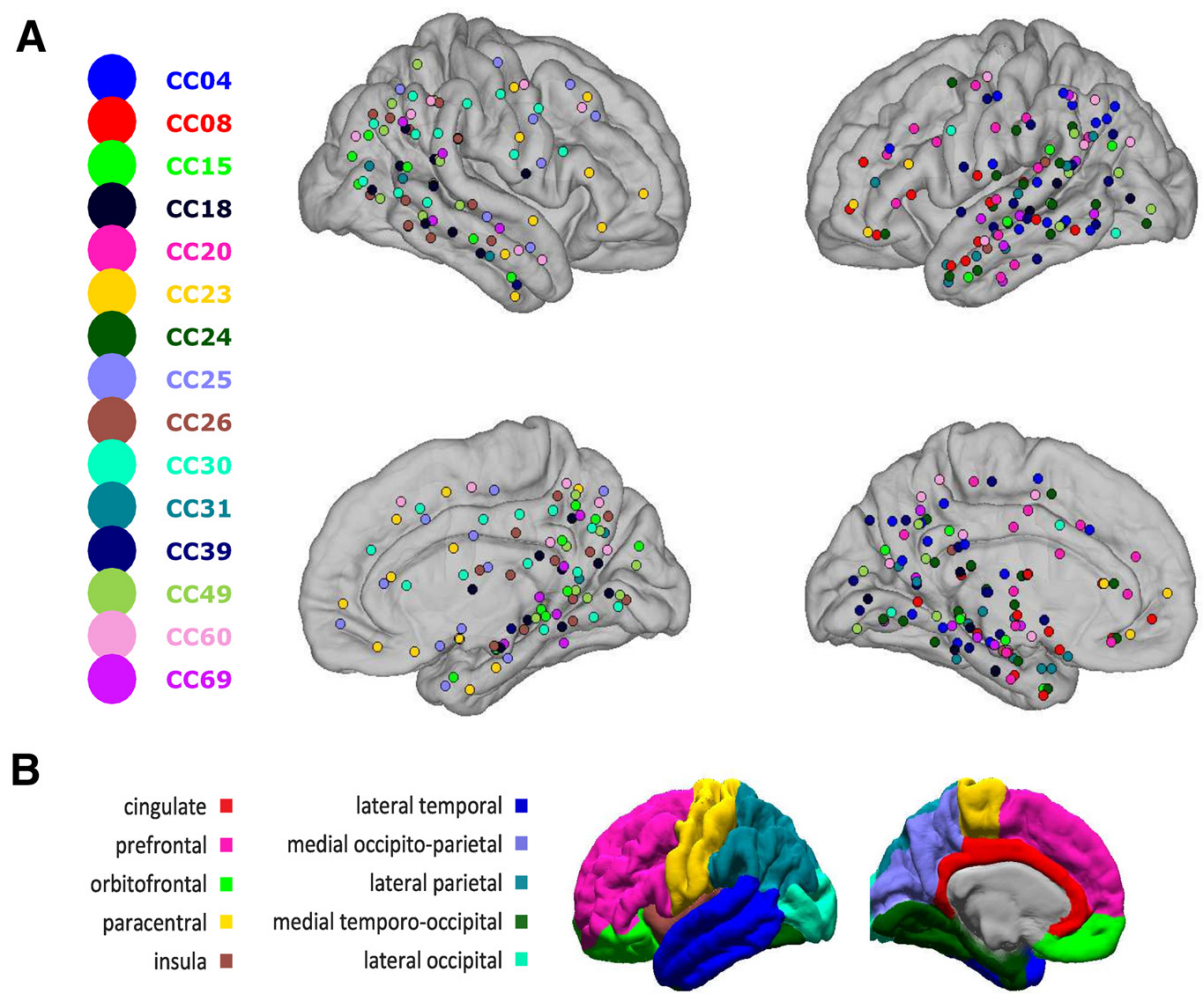
Electrode locations. ***A***, Anatomical locations of recording sites across 15 patients that are closest to the cortical and medial surfaces. Each dot location indicates the sEEG depth electrode entry point through the cortex or exit point through the medial view, therefore the locations are not exact as sEEG electrodes are not necessarily directly on the surface. Dot color indicates patient ID. ***B***, ROI anatomic map that was used to group channel pairs for analysis.

After implantation, electrodes were located by aligning postimplant CT to preoperative 3D T1-weighted structural MRI with 1-mm^3^ voxel size (Dykstra et al., 2012) using 3D Slicer (RRID: SCR_005619). The assignment of depth contacts to the anterior or posterior hippocampus was made with the posterior limit of the uncal head as boundary (Poppenk et al., 2013; Ding and Van Hoesen, 2015). The distance of each hippocampal contact from the anterior limit of the hippocampal head was obtained in FreeSurfer (RRID:SCR_001847). The CT-visible cortical contacts were then identified as previously described previously (Jiang et al., 2019) to ensure that activity recorded by bipolar transcortical pairs is locally generated (Mak-Mccully et al., 2017). Electrode contacts were rejected from analysis if they were involved in the early stages of the seizure discharge or had frequent interictal activity or abnormal spontaneous local field potentials (LFPs). FreeSurfer (Dale et al., 1999; Fischl et al., 1999a, 2004) was used to reconstruct from individual MRI scans the cortical pial and inflated surfaces, as well as automatic parcellation of the cortical surface into anatomic areas (Desikan et al., 2006), after a sulcal-gyral alignment process. Additionally, standard FreeSurfer regions of interest (ROIs) were combined into 12 composite ROIs (Fig. 1*B*), as well as different functional networks defined in (Rosen and Halgren, 2021) and adapted from network clustering on resting-state fMRI data (Ji et al., 2019). An average surface generated from previous work (Jiang et al., 2019) of 20 patients that included the 16 patients used in this study served as the basis of all 3D maps. While each cortical sEEG electrode contact’s location was obtained through direct correlation of CT and MRI as described earlier in this section, we obtained the cortical parcellation labels corresponding to each contact by morphing the right-anterior superior-oriented anatomic coordinates from individual surfaces to the average surface space (Fischl et al., 1999b). All visualizations were created with custom scripts in MATLAB 2016b (The MathWorks). For the majority of this study, we focus our analyses on the 12 ROIs in Figure 1*B*.

### Data collection and preprocessing

#### sEEG preprocessing and sleep stage detection

Continuous recordings from sEEG depth electrodes were made with a cable telemetry system (JE-120 amplifier with 128 or 256 channels, 0.016- to 3000-Hz bandpass, Neurofax EEG1200, Nihon Kohden) across multiple nights over the course of clinical monitoring for spontaneous seizures, with 1000 Hz sampling rate. sEEG channels were then referenced in a pair-wise manner with respect to neighboring electrodes. The neural signals were then notched filtered at 60 Hz and its harmonics to remove line noise. Bipolar channel pairs were rejected if they hit white matter only, display epileptic activity, or do not lie in the cortex or hippocampus. A total of 368 bipolar channel pairs across 15 patients were accepted for further analysis (Fig. 1*A*). One subject was rejected from further analyses because of the increased presence of artifacts in the sEEG signals (see below, Artifact rejection). The accepted bipolar channels were then filtered from 70–110 Hz using a zero-phase Chebychev Type II IIR bandpass filter to extract the high γ activity, which is shown to be highly correlated to neural spiking activity (Mukamel et al., 2005; Manning et al., 2009; Ray and Maunsell, 2011; Miller et al., 2014). The Hilbert envelope was obtained and then averaged across 1-min nonoverlapping windows to give an estimate of high γ activity. Resulting band-passed and averaged bipolar channels were visually inspected to reject channels with noisy HG (32 out of 368 channels were visually rejected). The same procedures were followed to investigate the effects of δ band mediation (see below, Correlation analyses), with the exception of applying a 1- to 4-Hz zero-phase Chebychev Type I IIR bandpass filter.

The total nonrapid eye movement (NREM) sleep durations vary across patients; beyond the normal variability (Carskadon and Dement, 2010), sleep may be disrupted in a clinical environment. Recordings were anonymized and converted into the European Data Format (EDF). Segmentation of patient NREM sleep/wake states from intracranial LFP alone was achieved by previously described methods using clustering of first principal components of δ-to-spindle and δ-to-γ power ratios across multiple LFP derived signal vectors (Gervasoni et al., 2004; Jiang et al., 2017) with the addition that separation of N2 and N3 was empirically determined by the proportion of DSs that are also part of slow oscillations (at least 50% for N3; Silber et al., 2007) since isolated DSs in the form of K-complexes are predominantly found in stage 2 sleep (Cash et al., 2009). Because of difficulty in distinguishing between REM sleep and waking in this dataset, only NREM stages were used in this study.

### Artifact rejection

A dual artifact rejection criterion was used on the neural signals to identify and remove outlier 1-min epochs. First, any 1-min epoch that exceeded 3 SDs above the channel high γ band mean for >20% of the channels was considered as artifactual and rejected (comprising 1.5 ± 1.5% of 1-min epochs across all patients and days). Additionally, for each minute, the power spectral density (PSD) was obtained to identify the presence of any peaks in the high γ band. We only accepted epochs for which the modulation in the high γ band presented itself as a broadband shift that is added to the 1/f noise characteristic of neural signals, rather than an oscillatory-like bump. Broadband shifts in the high γ band are most likely because of an overall increase in spiking activity (Mukamel et al., 2005; Manning et al., 2009; Ray and Maunsell, 2011; Miller et al., 2014), and it has been shown that asynchronous broadband shifts are linked to the fMRI BOLD response (Winawer et al., 2013). Narrowband peaks in the high γ band would be evidence of synchronized oscillatory behavior (Haller et al., 2018) or noise. For this study, we disregarded any epochs that displayed evidence of high γ oscillations or noise and only accepted epochs that show evidence of a broadband shift in power. To do this, we used a linear regression model to fit a line across the PSD calculated for each 1-min epoch across the 70- to 110-Hz frequency band. We then subtracted the fitted line from the PSD and look for the maximum value. If the max exceeded 3 dB across 20% of the channels, then that epoch is considered artifactual (10.8 ± 12% of 1-min epochs across all patients and days). Finally, any day’s worth of recording where the number of total artifacts exceeded 50% was removed from subsequent analyses (this resulted in a single subject being rejected from further analyses and no days rejected for the other subjects).

### Z-score normalization

For each day, the 1-min bipolar pairs were separated according to the sleep stage label assigned to that 1-min time bin. Then, the 1 min high γ amplitude estimate recorded by each bipolar pair in each sleep stage for each sleep period was z-scored with its mean and SD to arrive at the normalized high g activity (HGnorm). The same process was applied to the normalized high frequency component of the RR-interval. The HGnorm and HFnorm for each sleep stage were concatenated from each sleep period. This ensured that any linear correlations derived in subsequent analyses were because of within-sleep period and within-sleep stage variations, and not to a change in baseline that could happen across sleep periods, or across sleep stages within a specific sleep period. δ Band activity was also z-scored similarly.

### ECG preprocessing and HRV frequency-domain analysis

**Figure 2.**
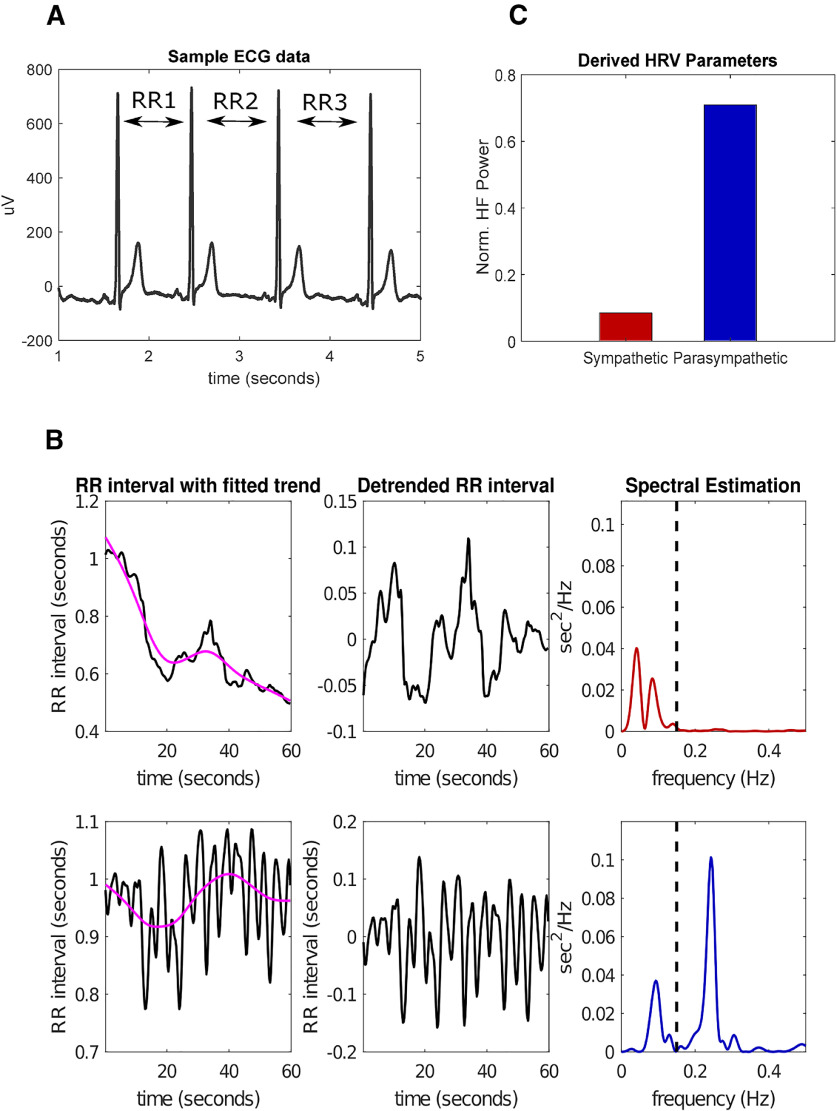
HRV calculations. ***A***, Sample 4-s ECG signal; the RR interval is determined from the time difference between subsequent R peaks. ***B***, Example calculations of HFnorm for two 1-min intervals, one with an overall sympathetic tone (top row) and one with an overall parasympathetic tone (bottom row). The very LF trend (magenta line) is removed from the raw RR intervals (left-most plots), resulting in the detrended time series (middle plots). PSD estimates show that most of the power is in the “HF” (0.15–0.4 Hz) for the parasympathetic interval (right-most plots). ***C***, Calculated normalized HF power for the two intervals displayed in Figure 2*B*. The bar colors correspond to the matching colors in the spectral estimation plots.

ECG recordings were also acquired throughout the days in which the patient was in the hospital. The ECG recordings were visually inspected and artifacts were rejected if the raw value of the recording exceeded 3 SDs above the median, which indicates a movement artifact (0.34 ± 0.67% across all patients and days). Furthermore, the ECG recordings were analyzed initially using the Kubios Premium HRV software (Tarvainen et al., 2013) to detect the QRS wave and pinpoint the location of the R peaks (Fig. 2*A*). Then, the RR interval was generated by finding the time between adjacent R peaks, and artifacts were corrected for missed and ectopic beats using an automatic artifact rejection algorithm (Lipponen and Tarvainen, 2019). The RR interval was then interpolated using a cubic spline interpolation and then sampled at 4 Hz to ensure even sampling of the signal, as this enabled us to extract accurate frequency-domain metrics from the data.

The resulting interpolated RR interval was then segmented into 1-min bins and detrended using the smoothness priors algorithm that removed very low frequency (LF; <0.035 Hz) and nonstationary trends in the RR interval, which could distort frequency domain analyses that require the signal to be stationary (Tarvainen et al., 2002; Fig. 2*B*). The mechanisms that drive the very LF component of the RR signal could be because of thermoregulatory cycles or changes in plasma renin activity, however, the mechanisms driving these slow oscillations are not well understood and therefore removed from any subsequent frequency domain analyses (Berntson et al., 1997).

We then calculated the PSD of each detrended 1-min segments of RR intervals using Welch’s method with 30-s windows and 75% overlap. We used a hamming window to prevent spectral leakage. The power in the LF band, from 0.04 to 0.15 Hz, and the high-frequency (HF) band, from 0.15 to 0.4 Hz of the RR interval are calculated by estimating the area under the PSD in those bands using the trapezoidal method. The LF band is primarily modulated by both the sympathetic and parasympathetic nervous systems. On the other hand, the HF band is modulated by the parasympathetic nervous system and mainly through respiratory sinus arrhythmia (Shaffer and Ginsberg, 2017). Once the power of each of the two bands was calculated (Fig. 2*B*), we estimated the normalized HF component (HFnorm) by dividing the HF power by the total power in the two bands HF/(LF+HF) (Fig. 2*C*). The HFnorm reflects the proportion of parasympathetic to sympathetic activity and is used as a marker of sympathovagal balance (Heathers, 2014). A higher HFnorm value indicates a shift of the ANS toward a parasympathetic state. As mentioned earlier, the HFnorm is z-scored to remove sleep stage and sleep period related effects. We refer to the z-scored HFnorm values as HFnorm moving on for simplicity.

### Experimental design and statistical analysis

#### Correlation analyses

For each patient, we calculated the Spearman correlation coefficient (*r*) of the HGnorm with the HFnorm for each bipolar pair in each NREM sleep stage. To convert the correlation coefficients to a standard normal distribution for subsequent hypothesis tests, we applied the Fisher z-transformation on the Spearman correlation coefficients to derive z-score values, z. A bipolar pair is labeled as significantly correlated in each sleep stage if their *p* value determined from the Spearman correlation analysis is <0.05, after applying a false discovery rate (FDR) correction for the number of bipolar pairs in the region where it is located. We then pooled together all of the Fisher z-transformed correlation coefficients $\left(z\right)$for all channels in their defined ROI, both the significant and unsignificant bipolar pairs. To determine whether there is an overall patient-wide correlation between the HGnorm and HFnorm in each ROI/sleep stage, we applied a Student’s *t* test to evaluate the null hypotheses that the mean of the pooled z values in each ROI and sleep stage does not deviate from zero. Additionally, for each bipolar pair, we calculated the partial Spearman correlation between the HGnorm and the HFnorm conditioned on the δ band activity (partialcorr function in MATLAB) and subsequently applied the Fisher z-transformation. We reapplied the Student’s *t* test to determine whether the pooled partial correlation z values deviate from zero for each ROI/sleep stage. This experimental design investigates whether the overall correlation between HG and HRV is mediated by the δ band since studies have shown that δ band up states and DSs modulate HG in humans during sleep (Csercsa et al., 2010; Halgren et al., 2018).

#### Bootstrapped percentage of correlated channels analysis

Within each ROI and sleep stage, there could be bipolar pairs where their HGs are either very positively or negatively correlated with HRV. Therefore, it is important to not only analyze the overall mean correlation, but the overall percentage of statistically correlated channels in each ROI. A ROI could have a large percentage of statistically correlated channels with autonomic modulation and at the same time have mean correlation coefficient to not statistically deviate from zero. For each sleep stage, we calculated the proportion of statistically significant channels in each ROI as a measure of the undirected connectivity between the ANS and the corresponding ROI. Since there exists a variable number of samples in each ROI (because of the different distribution of bipolar pairs across subjects), the *p* value comparison between different regions would be unfair as *p* value is correlated with the number of datapoints in a correlation analysis. To alleviate this issue, we applied a bootstrapped analysis by randomly subsampling the HGnorm from each bipolar channel pair and the HFnorm with replacement and then repeating the Spearman correlation analyses described in the previous section. We set the number of samples in each correlation analysis to be equal (*n* = 50) to ensure that an uneven number of samples did not influence the correlation *p* values. We repeated the correlation analyses for a total of *N* = 1000 iterations, and in each iteration, we calculated the percentage of statistically correlated bipolar channel pairs in each ROI and sleep stage separately (FDR corrected with α = 0.05). This generated a distribution of the percentage of channels that have a statistically significant correlation in each ROI and sleep stage combination between high γ activity and autonomic tone (for both the high γ band correlation and the partial correlation mediated by δ band). We then determined the average percentage of correlated channels at each ROI and sleep stage combination by averaging the distributions of the percentage of statistically significant bipolar pairs.

### Statistical analysis

For each of the statistical tests, we used an FDR correction with α = 0.05 (Benjamini and Hochberg, 1995). The FDR correction procedure was implemented as follows: for a total of N hypotheses, each with corresponding *p* values Pi (i ∈ N), which are sorted in ascending order to identify Pk [k being the largest i for which Pi ≤ (i/N) * α], all hypotheses with *p* values less than or equal to Pk would be rejected. We used the Student’s *t* test to determine whether the mean of the overall correlation in each ROI/sleep stage deviated from zero. In the bootstrapped percentage of correlated channels analysis, the FDR correction to determine the percentage of statistically significant channel pairs in each iteration was applied on each ROI separately, therefore, the number of comparisons is the number of channel pairs in each ROI. The FDR correction for the Student’s *t* tests was applied for each correlation measure separately for a total of 36 comparisons per analysis (12 regions, three sleep stages).

To investigate whether the overall patient-wise correlation in each ROI is affected by the sleep stage, we applied an *N*-way ANOVA using the ROI and sleep stage as the grouping variables and the Spearman correlation coefficient as the response variable. Finally, to determine whether the overall patient wise correlation is because of patient-specific variation, we fit and compared linear mixed-effects models. Specifically, we compared an LME model that incorporated the ROI and Sleep stage as predictor variables with a simplified model that only used the patient ID as a predictor variable. In the bootstrapped percentage of correlated channels analysis, we applied a one-way ANOVA using the mean of the percentage of correlated channels as the response variable and sleep stage as the grouping variable to determine whether sleep stage has a significant effect on the ANS-CNS undirected connectivity.

Finally, we tested whether the direction of the correlation between HGnorm across hippocampus and cortex and HFnorm is consistent in sleep stage transitions (agnostic of location). We first selected the channels where HGnorm and HFnorm were determined to be statistically correlated, as separately tested in each of the sleep stages. Then, we determined the number of channels that have the same direction of correlation across all sleep stages, i.e., positive correlations in N1, N2, and N3, or negative correlations in N1, N2, and N3. In this analysis, if we assume that each channel’s HGnorm has an equal probability of being either positive or negatively correlated with HFnorm, then the probability that that channel has the same correlation direction for all sleep stages is 0.25. We then applied a binomial test with the probability of success being 0.25 to determine whether a statistically correlated channel is more likely to have the same direction of correlation across all sleep stages or not.

## Results

### Summary of data

A total of 336 sEEG sites across the 15 patients were included for further analysis (for demographic and clinical information, see Table 1). Figure 1*A* shows the distribution of cortical penetration sites of the sEEG shanks. There is broad coverage across multiple cortical sites in both left and right hemispheres. Figure 1*C* shows the mapping of neocortical ROIs that we apply to cluster the sEEG site locations for further analyses. Anterior and posterior hippocampus, as well and lateral temporal and parietal lobe coverages, is present in the most number of subjects (>11). Also, there is coverage in cingulate and insular cortices, as well as lateral-occipital, paracentral, prefrontal, medial occipito-parietal, and medial temporo-occipital lobes. For each ROI, we have at least 5 subjects with coverage in that ROI. Across multiple days, we collected on average (mean ± SD) 170 ± 98 min for N1, 526 ± 295 for N2, and 328 ± 215 min for N3, for a total of an average of 1024 min (∼17 h) of sleep data per patient. We subtrated the patient-specific mean of the HFnorm to investigate sleep stage dependent shifts, and then applied a one-way ANOVA using the baseline HFnorm as a response variable and sleep stage as a grouping variable. Sleep stage has a significant effect of HFnorm, *F*_(2,42)_ = 11.28, *p* = 0.0001. The Gaussianity of the residuals was tested using the one-sample Kolmogorov–Smirnov test and we could not reject the hypothesis that the residuals come from a normal distribution (*D*_(45)_ = 0.11, *p* = 0.57). We then applied a *post hoc* analysis using Tukey’s range test and found that baseline HFnorm in N3 is greater than N1 and N2 (*p* = 0.0001 and *p* = 0.042 for N3 vs N1 and N3 vs N2, respectively). This indicates a shift toward parasympathetic balance when entering deeper sleep stages, as a previous study has shown in healthy individuals (Elsenbruch et al., 1999). The average HFnorm across patients are 0.52, 0.55, and 0.59 for N1, N2, and N3, respectively. These values were similar to previous work investigating the HFnorm in healthy individuals during sleep (Busek et al., 2005). Similarly to our investigation of baseline HFnorm,. we applied the same approach to the HG and found a significant effect of sleep stage, *F*_(2,42)_ = 14.48, *p* = 1.65 × 10^–5^. *Post hoc* analyses using Tukey’s range test found that baseline high γ activity across patients in N3 is lower than N1 and N2 (*p* = 1.172 × 10^–5^ and *p* = 0.0036 for N3 vs N1 and N3 vs N2, respectively). We tested for the Gaussianity of the residuals using the one-sample Kolmogorov–Smirnov test and could not reject the hypothesis that the residuals come from a normal distribution (*D*_(45)_ = 0.071, *p* = 0.9668).

**Table 1:** Patient and recording information

Patient	Age (mean ± SD)	Sex	Handedness	Number of channels	Number of sleep periods	Number of 1-min samples		
						NREM1	NREM2	NREM3
CC04	20	M	Right	20	3	141	178	175
CC08	58	F	Right	28	3	164	998	88
CC15	42	M	Left	24	4	159	148	497
CC18	18	F	Left	28	3	142	197	246
CC20	22	M	Left/right	18	3	150	297	336
CC23	40	F	Right	16	5	154	588	879
CC24	43	F	Right	18	3	202	219	223
CC25	16	M	Right	16	5	144	504	536
CC26	32	F	Right	35	3	91	507	197
CC30	36	M	Left	28	4	162	820	420
CC31	21	F	Left	21	3	105	922	121
CC39	21	F	Right	18	8	472	805	577
CC49	29	F	Right	23	4	279	351	226
CC60	24	M	Right	22	3	56	492	170
CC69	31	F	Right	21	4	120	886	234
Mean ± SD	30.2 ± 11.8			21 ± 7.6	3.6 ± 1.5	170 ± 98	526 ± 295	328 ± 215

### Overall correlation effect is present in multiple sites and dependent on NREM sleep stage

**Figure 3.**
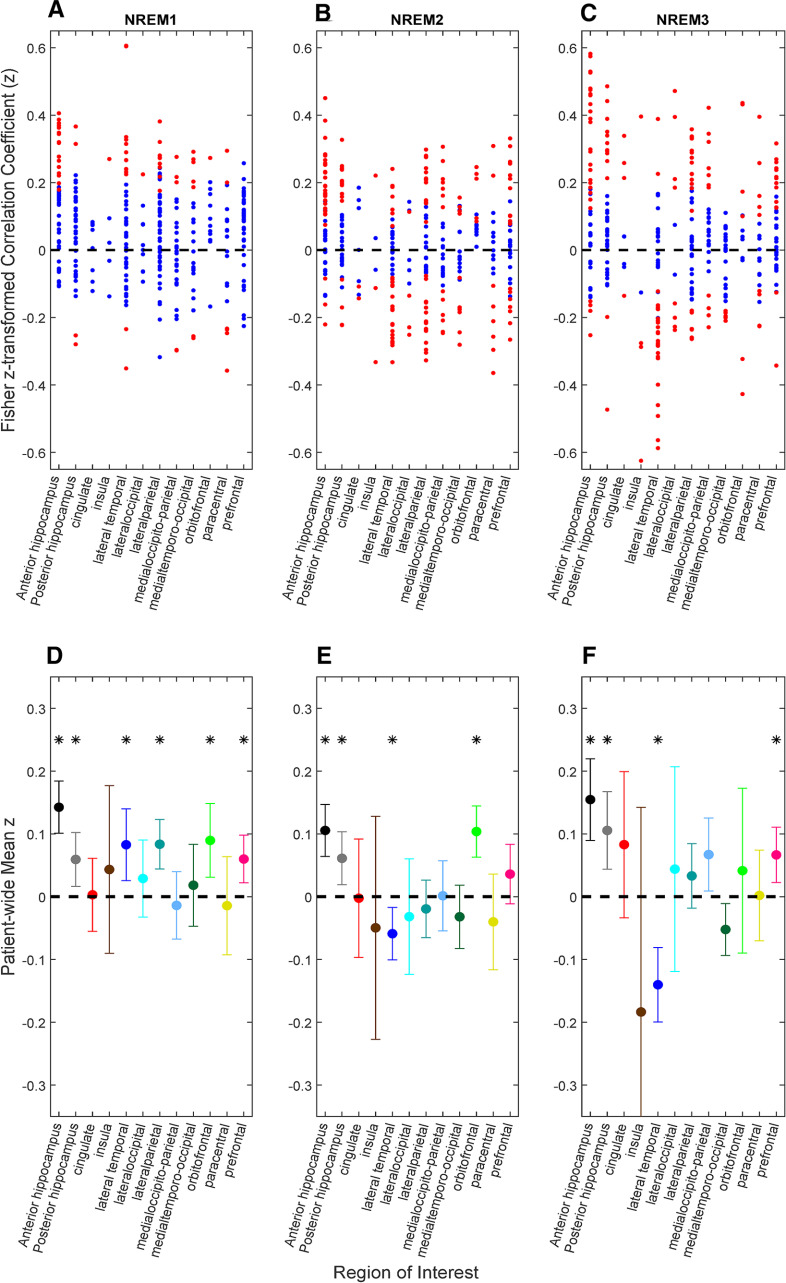
Correlation of population neural activity (HGnorm) and estimated parasympathetic balance (HFnorm). The distribution of the Fisher z-transformed correlation coefficient z values for each ROI in each sleep stage is displayed in the topmost plots (***A–C***), The red dots are statistically significant channel pairs, whereas the blue dots are not (FDR corrected *p* < 0.05). The mean and 95% confidence interval of the z values are displayed in panels ***D–F***. Regions where the mean z is significantly differ from zero are labeled with an asterisk. Each cortical region is color coded according to Figure 1*B*.

Figure 3*A–C* shows the distribution of z values derived from the Fisher z-transformed Spearman correlations between the 1-min averaged HG estimate (HGnorm) with the normalized HF power of the RR intervals (HFnorm). The bipolar pairs labeled as statistically significant in each ROI and sleep stage are shown in red. In total, 20.1%, 50.1%, and 46.7% of the bipolar pairs across patients are significantly correlated in N1, N2, and N3, respectively. When applying the FDR correction across ROIs, the percent of correlated bipolar pairs were 19.6%, 51.5%, and 46.4% for N1, N2, and N3, respectively. It is evident that there is a wide range of effects for each ROI across all NREM sleep stages as the z values range from −0.62 to 0.60. However, it is also evident that in some ROIs, the distribution of z values is consistently either greater than or less than zero. Positive correlations suggest that increased neural activity in a bipolar pair is associated in with an overall shift of the ANS toward a parasympathetic state, and conversely, negative correlations suggest that increased neural activity is associated in that bipolar pair with an overall shift of the ANS toward sympathetic state.

The overall interpatient trends are summarized in Figure 3*D–F*. ROIs for each sleep stage that have an overall mean z that is statistically different from zero are indicated with an asterisk. The mean Fisher z-transformed correlation coefficients and *p* values generated from the Student’s *t* tests (to test whether the mean of the population is statistically different from zero) for each ROI/NREM combination are listed in Table 2. Both the anterior and posterior hippocampus show an overall positive trend in all NREM stages. Additionally, there is an overall statistically significant positive trend in lateral temporal, lateral parietal, orbitofrontal and prefrontal lobes in N1, orbitofrontal lobe in N2, and prefrontal lobe in N3. Higher neural activity in these regions is directly correlated with a shift to a parasympathetic autonomic state, with each region showing a profound effect according to the sleep stage. Finally, the lateral temporal lobe shows an overall negative trend across patients in N3 that is statistically significant (mean *z* = −0.14, *t*_(45)_ = −4.64, *p* = 3 × 10^–5^). Overall, of the 12 regions and three states, 14 means were significantly different from zero, 12 in the positive direction, and two in the negative, indicating that greater neural activity is more often associated with parasympathetic tone (binomial test, one-tailed, *p* = 0.0065). To ensure that the mean in each ROI in each sleep stage is not deemed as statistically significant because of deviations from nonnormality when using the Student’s *t* test, we reapplied the statistical analysis using the nonparametric Wilcoxon signed rank test that tests whether the median of a distribution is zero, with the only assumption being that it is continuous and symmetric around the median. Our results did not differ in comparison to applying the Student’s *t* test. When applying the same correlation analysis using the functional network labels instead of the structural ROIs, we observe that a statistically significant overall correlation is only present in N1 for the default mode (mean *z* = 0.088, *t*_(87)_ = 4.58, *p* = 1.5 × 10^–5^) and frontoparietal networks (mean *z* = 0.065, *t*_(45)_ = 3.3, *p* = 0.0019). In comparison to waking studies (Beissner et al., 2013), our work shows that the DMN is not a major contributor to any autonomic modulation in deeper sleep stages. The mean Fisher z-transformed correlations examined in this analysis identifies the existence of a patient-wide trend relating high γ activity and autonomic tone.

**Table 2:** Average correlation of high γ and HRV within region

Location	Number of bipolar channels	Number of patients	Sleep stage 1		Sleep stage 2		Sleep stage 3	
			Mean z [95% CI]	*p* value	Mean z [95% CI]	*p* value	Mean z [95% CI]	*p* value
Anterior hippocampus	48	12	0.14 [0.1, 0.18]	2.2 × 10^-8^	0.11 [0.064, 0.15]	8.2 × 10^-6^	0.15 [0.089, 0.22]	2.6 × 10^-5^
Posterior hippocampus	39	11	0.059 [0.016,0.1]	0.01	0.061 [0.019, 0.1]	0.0072	0.11 [0.044, 0.17]	0.0019
Cingulate	8	6	0.003 [−0.055, 0.061]	0.92	−0.0024 [−0.097,0.093]	0.96	0.083 [−0.033, 0.2]	0.21
Insula	5	4	0.043 [−0.09, 0.18]	0.56	−0.049 [−0.23, 0.13]	0.62	−0.18 [−0.51, 0.14]	0.33
Lateral temporal	46	13	0.083 [0.026, 0.14]	0.0068	−0.059 [−0.1, −0.017]	0.0081	−0.14 [−0.19, −0.081]	3 × 10^-5^
Lateral occipital	10	6	0.029 [−0.032,0.09]	0.38	−0.032 [−0.12, 0.06]	0.52	0.044 [−0.12, 0.21]	0.61
Lateral parietal	52	14	0.084 [0.044, 0.12]	0.0001	−0.019 [−0.065, 0.026]	0.41	0.033 [−0.018, 0.084]	0.21
Medial occipito-parietal	28	11	−0.014 [−0.067, 0.04]	0.62	0.0014 [−0.054,0.057]	0.96	0.067 [0.0089, 0.13]	0.032
Medial temporo-occipital	24	10	0.018 [−0.047, 0.083]	0.59	−0.032 [−0.083, 0.018]	0.23	−0.052 [−0.094, −0.011]	0.022
Orbitofrontal	13	5	0.089 [0.031, 0.15]	0.011	0.10 [0.063, 0.14]	0.0003	0.041 [−0.09, 0.17]	0.55
Paracentral	19	9	−0.014 [−0.092, 0.063]	0.73	−0.04 [−0.12, 0.036]	0.32	0.002 [−0.07, 0.074]	0.97
Prefrontal	38	8	0.060 [0.022, 0.097]	0.0037	0.036 [−0.011, 0.083]	0.14	0.067 [0.025, 0.11]	0.0053

The correlation (r) was calculated between the normalized high γ and HRV measures (HGnorm and HFnorm) within each sleep stage, and averaged across all channels within each ROI. The Student’s *t* test was used to determine whether the mean Fisher z-transformed rvalues (z) differs from zero (FDR corrected *p* < 0.05).

Furthermore, we applied a two-way ANOVA to determine whether the correlation between neural activation and autonomic balance shows a main effect of anatomic location, sleep stage, or their interaction. The ANOVA analysis shows that there is a main effect of both anatomic location (*F*_(12,969)_ = 9.24, *p* = 4.6 × 10^–17^) and sleep stage (*F*_(2,969)_ = 3.98, *p* = 0.018) on the correlations, as well as the interaction between location and sleep stage (*F*_(24,969)_ = 2.38, *p* = 0.0002). We tested the Gaussianity of the residuals generated from the ANOVA model using the one-sample Kolmogorov–Smirnov test and could not reject the null hypothesis that the residuals come from a normal distribution (*D*_(1008)_ = 0.0174, *p* = 0.9156). The ANOVA test shows that there exists a global effect on sleep stage across all regions and that the autonomic networks across cortex on hippocampus is dependent on sleep stage. Mainly, the overall correlation across all sites in N1 is higher than N2 (*post hoc* Tukey’s range test, *p* = 0.015).

Finally, to take into account patient wide variability, we fitted two linear mixed-effects models on the data. First, we fitted a simplified model that only takes into account the patient’s ID as a random effect, Corr ∼ 1 +(1|Patient_ID). This model would be appropriate if the patient-specific variability is the main factor that contributes toward the correlation values. The model we compare it to is a more complex model that takes into account anatomic location and sleep stage and their interaction as fixed effects and the patient ID as the random effect, Corr ∼ 1 + AL*SS + (1|patient_ID). Using a theoretical likelihood ratio test, we found that indeed the more complex model is a better fit to the data that the simplified model (χ^2^_(38)_ = 184.5, *p* = 3.91 × 10^–21^). Along with the two-way ANOVA analysis, this provides further evidence that there is an apparent effect of the anatomic location, sleep stage, and their interaction on the overall correlation between the HGnorm and the HFnorm.

Next, we investigated whether the direction of correlation of correlated channels is consistent across sleep stages. Thirty channels were found to be statistically correlated across all sleep stages, and 28 of those 30 channels have the same direction of correlation across all sleep stages. We then applied a binomial test to determine whether a statistically correlated channel is more likely to have the same direction of correlation across all sleep stages. In the binomial test, the probability of success (success being the direction of correlation across all sleep stages is the same) is 0.25, the number of trials is 30, and the number of successful trials is 28. The likelihood that a bipolar pair exhibits the same direction of correlation across all sleep stages is higher than chance (one-tailed binomial test, *p* = 3.44 × 10^–15^). Thus, although the correlation between HGnorm and HFnorm could be either positive or negative, in 93% of the channels the direction was consistent across stages.

Previous studies have observed that there exists a heartbeat evoked potential (HEP) in intracranial electrophysiology (Kern et al., 2013; Park et al., 2018; Park and Blanke, 2019). The HEP is also present in high γ band (Kern et al., 2013). One concern is that our results could be driven by the HEP and not be related to autonomic modulation. Increase in heart rate and blood flow could also mechanically displace the channels, which could generate an artificial response and induce a systemic bias in our results. To test whether the HEP or heart-rate-related confounds are driving our results, we reapplied the correlation analysis using the normalized mean heart rate (HRnorm) rather than the HFnorm, using the same z-scoring technique described in Materials and Methods. We did not find statistically significant interpatient trends in any ROI/sleep stage. Further, we used the mean heart rate as a mediating variable and calculated the partial correlation between the HGnorm and HFnorm. The number of statistically significant ROIs across all sleep stages is the same as our previous analysis looking at correlation between HGnorm and HFnorm. These two control analyses provide further evidence that our result is driven by autonomic modulation and not by HEP or heart-rate generated artifacts.

### Correlation of high γ band with the autonomic response is related to δ band activity

**Figure 4.**
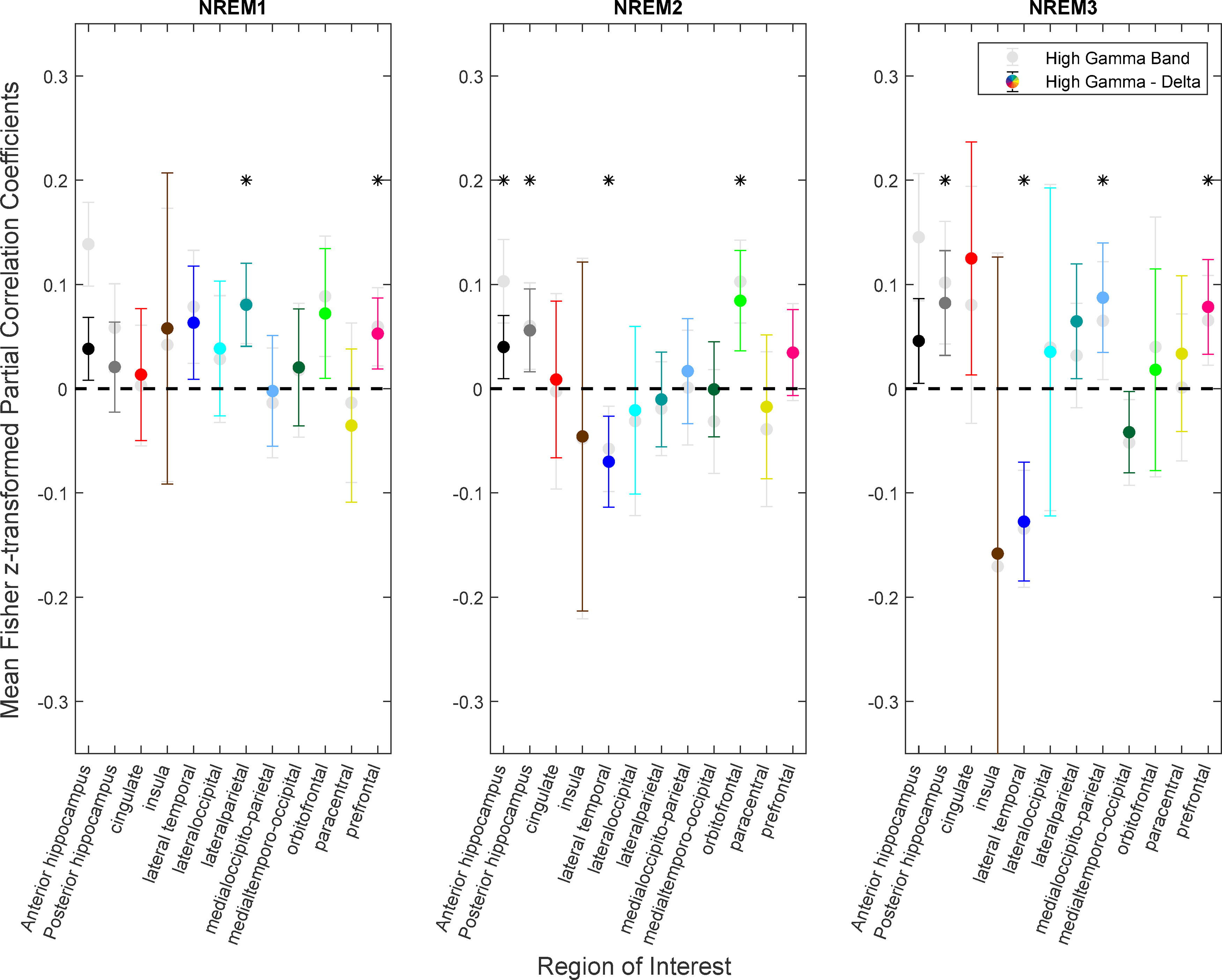
Correlation of population neural activity and autonomic state after conditioning on δ. Similarly to Figure 3*D–F*, the means and 95% confidence intervals of the z values calculated from the correlation between HGnorm and HFnorm but after conditioning on δ band activity and applying the Fisher z-transformation on the correlation coefficients. Color coding of cortical ROIs are equivalent to Figure 1*B*. The unconditioned mean and 95% confidence intervals shown in Figure 3*D–F* are superimposed in light gray to facilitate their comparison. Regions where the mean z is significantly differ from zero are labeled with an asterisk.

NREM stages N2 and N3 are characterized by high levels of δ band activity, which powerfully modulates high γ activity in humans (Csercsa et al., 2010; Halgren et al., 2018), raising the possibility that the correlations found between high γ band and HFnorm are mediated by variations in δ activity within each sleep stage. Figure 4 shows the Fisher z-transformed partial correlation of the HGnorm with HFnorm conditioned on the δ band activity estimate for each anatomic location and sleep stage combination. Overall, the number of statistically significant locations was lower (from *n* = 14 to *n* = 10, across N1, N2, and N3) when removing the effect of δ band activity variation. A key difference is a decrease in anterior hippocampal Fisher z-transformed correlation coefficients when the effects of δ band activity were removed in N1 and N3. The other sites becoming statistically significant or insignificant had only minimal increases or decreases in the absolute value of the average Fisher z-transformed correlation coefficients, z. We conclude that the overall pattern of correlations in the cortex is related but mildly affected by variations in δ band activity. Studies have shown the presence of θ-high γ phase amplitude coupling in REM sleep in mice (Scheffzük et al., 2011; Bandarabadi et al., 2019) and increase in θ rhythm during sleep onset in humans (Tobaldini et al., 2013). Therefore, as a further control, we applied the same analysis while correcting for θ band (5–8 Hz) influences. The number of statistically significant ROIs across all sleep stages was lower in comparison to applying to correlation analysis without mediating for θ band (from *n* = 14 to *n* = 9). However, anterior hippocampus and lateral temporal lobe still exhibits a significant effect across all sleep stages when conditioned on θ band activity.

### Percentage of correlated channels between high γ activity and autonomic tone generally increases during deep sleep

In Figure 3*A–C*, as for almost all regions and all sleep stages, it is evident that within each anatomic location, some channel pairs across patients show a negative correlation, while others show a positive correlation. A ROI could not exhibit an effect when only looking at the patient wide average correlation, but exhibit an increase in the percentage of correlated channels between the CNS-ANS. Therefore, it is essential to not only investigate whether the overall patient wide correlation was statistically different from zero but also the strength of the CNS-ANS undirected connectivity measured by the percentage of statistically correlated channel pairs in each anatomic location and sleep stage.

**Figure 5.**
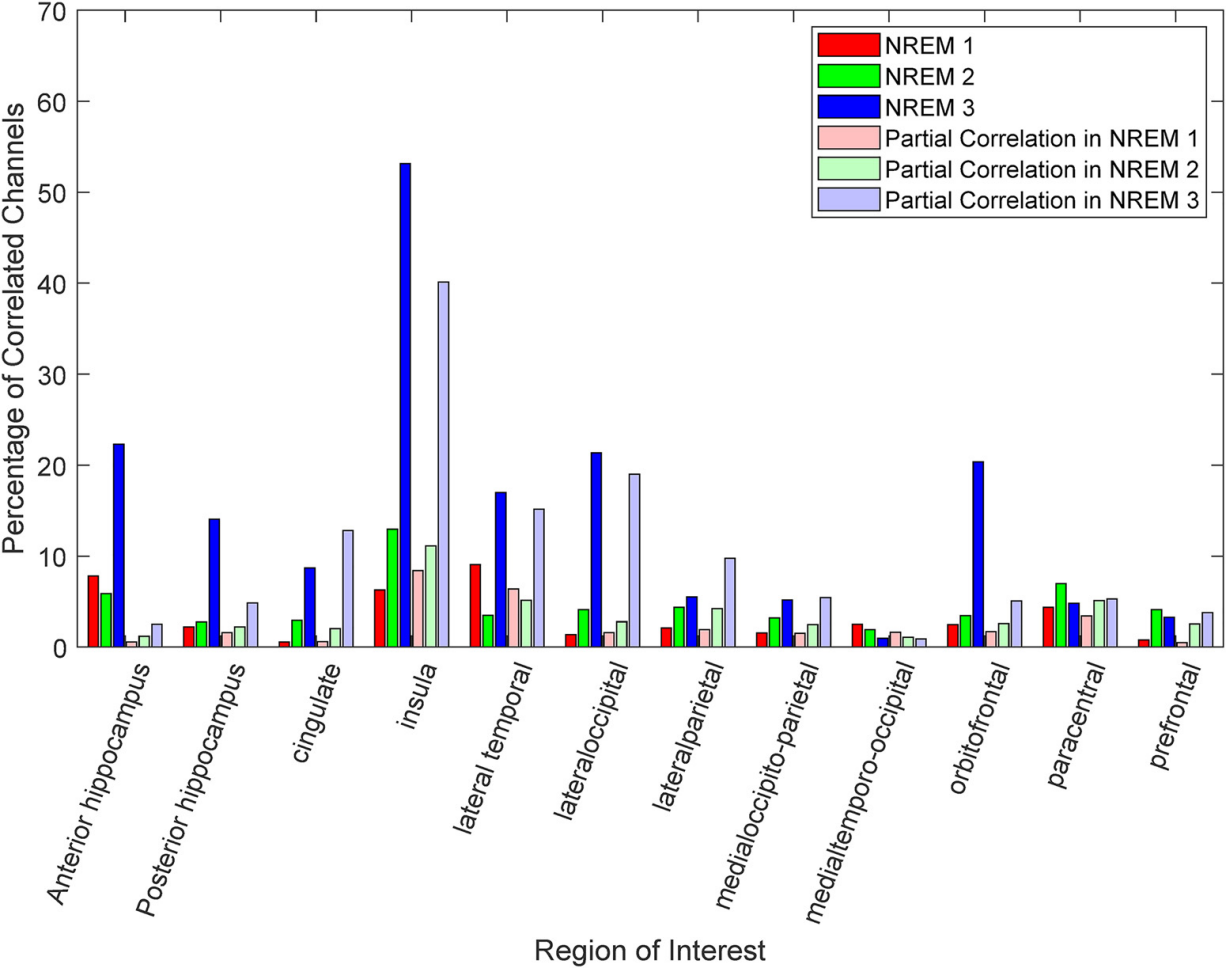
Distribution of responsive channels across locations and sleep stages. The percentage of channels where the correlation of HGnorm and HFnorm is statistically significant in each ROI is plotted for each sleep stage, with conditioning on δ activity (light colors) and without (dark colors). Percentage values for each ROI/sleep stage/correlation measure are shown in Extended Data Table 5-1.

First, a key finding in Figure 5 is that for many anatomic locations, the overall percentage of statistically correlated channel pairs increases in N3 as compared with N1 and N2, indicating that there is a stronger CNS-ANS undirected connectivity in deeper sleep. This was verified by applying a one-way ANOVA using the mean of the percentage of correlated channels as the response variable and sleep stage as the grouping variable. Sleep stage has a significant effect on the percentage of correlated channels between ROIs and autonomic tone (*F*_(2,33)_ = 6.24, *p* = 0.005). A *post hoc* Tukey’s range test confirms that the percentage of correlated channels in N3 is greater than both N2 (*p* = 0.019) and N1 (*p* = 0.0078). This phenomenon is mainly observed in both anterior and posterior hippocampus, insula, lateral temporal, lateral occipital, and orbitofrontal lobes, with the insula having the largest percentage of correlated channels in N3 at 53.1% of channels (bootstrapped) having a statistically significant correlation, although the insula did not show a statistically significant positive or negative trend in the previous analyses. This indicates that within the same region of the brain, statistically significant correlations between the autonomic system and neural activity could be in opposite directions.

10.1523/ENEURO.0194-21.2021.tab5-1 Extended Data Table 5-1 **The percentage of correlated channels (bootstrapped) using the mean correlation and partial correlation in different sleep stages for each ROI**. Download Table 5-1, DOCX file.

Figure 5 also displays the percentage of partially correlated (δ band conditioned) channel pairs in the lighter colors. The percentage of correlated channels in both the anterior and posterior hippocampal after removing the effect of δ band dropped significantly, most notably in N3, from 22.3% to 2.5%, respectively ( $Wilcoxonsignedranktest,Z=27.0,p=1.6\times 10^{-160}$) and 14.1% to 4.8% ( $Z=26.0,p=2.7\times 10^{-149}$). The percentage of correlated channels in insula and orbitofrontal dropped as well in N3, from 53.1% to 40.1% $(Z=9.95,p=2.4\times 10^{-23}$) and 20.4% to 5.1% $(Z=23.65,p=9.6\times 10^{-124}$), respectively. The cingulate, as well as the lateral parietal lobe, show a small increase in the percentage of correlated channels in N3 when applying the partial correlation from 8.7% to 12.8% $(Z=-12.48,p=1\times 10^{-35}$) and 5.5% to 9.8% $(Z=-29.65,p=1.1\times 10^{-94}$), respectively. Other anatomic locaitons showed little to no change in the percentage of correlated channels (<2.5% after conditioning on δ band). We reapplied the one-way ANOVA analysis on the percentage of partially correlated channels and found that sleep stage still has a significant effect on the ANS-CNS undirected connectivity even after δ band mediation (*F*_(2,33)_ = 5.06, *p* = 0.0121). The values of the mean correlation and partial correlation percentage of correlated channels for all ROIs, sleep stages, and correlation measures are shown in Extended Data Table 5-1.

## Discussion

In this study, we have collected and analyzed ECG and neural sEEG recordings from 15 subjects during sleep and quantified the relationship in terms of average linear correlations and percentage of correlated channels between HG in 12 distinct cortical and hippocampal locations and autonomic tone as indexed by the normalized HF component of the heart rate. To our knowledge, this is the first attempt to understand the brain-heart interaction during sleep using intracranial recordings. We have found a significant trend in the correlation between the high γ and autonomic tone in distinct anatomic locations across multiple patients that differ according to the sleep stage. In some locations (e.g., the anterior hippocampus in all sleep stages as well as the orbitofrontal cortex in N2), these correlations trended positive, indicating increased activity during a higher parasympathetic tone. In others (e.g., the lateral temporal lobe in N3), the correlation trended negative, suggesting higher cortical activity during a greater sympathetic tone. However, the direction of the correlation varied across the electrodes in each structure, indicating a significant but variegated response. Overall, predominantly positive correlations were more common, and a global response was evident as a function of sleep stage.

**Figure 6.**
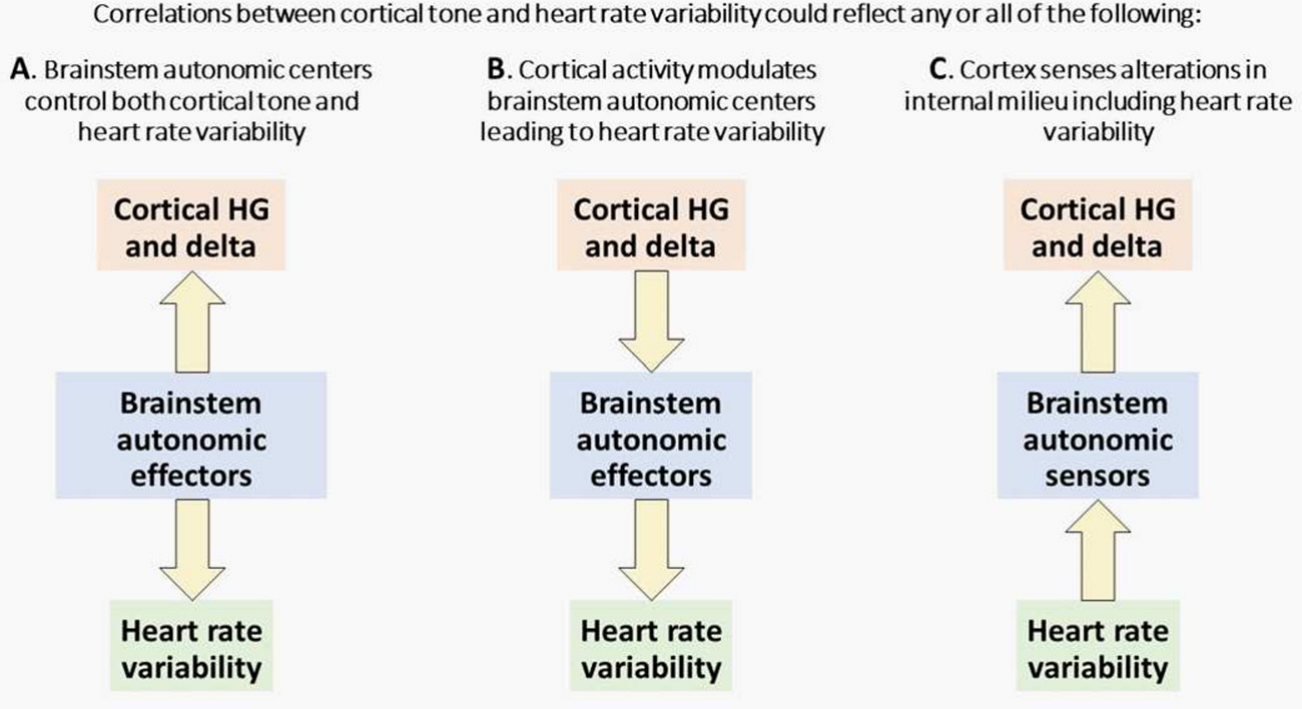
Possible functional relationships underlying the correlation between cortical and hippocampal activity and HRV during sleep. Panels ***A–C*** show the possible causal relationships between cortical activity, heart rate variability, and brain stem autonomic effectors that could explain the results shown in this work.

Although the neural activity in many bipolar channels was strongly correlated with autonomic tone, the population level mean correlation coefficient does not exceed an r absolute value of 0.15. The overall correlation identifies a trend that is patient-wide, as many bipolar channels in a region across patients can have either a positive or negative correlation of high γ activity and autonomic tone. A previous study also shows that on a population level, the correlation coefficients between neuronal firing rate and cardiac cycle duration is within the same range as found in this work (Kim et al., 2019). These correlations could serve many roles during sleep (Fig. 6): (A) modulation by brainstem autonomic efferents of both cortical/hippocampal tone and HRV, as part of an overall modulation of all internal organs, including the brain and heart; (B) control by cortical/hippocampal regions of brainstem autonomic centers which project to the heart; and/or (C) viscerosensory responses by cortical/hippocampal regions to changes in the internal milieu. Indeed, there is ample anatomic and physiological evidence for all three functional relationships, and they may all be reflected in the current results. Overall, our results demonstrate and characterize the relationship between visceral and cortical/hippocampal state.

We only found two functional networks in N1 that had a significant effect when investigating the correlation between HGnorm and HFnorm. Previous studies have theorized that the DMN is a major regulator of parasympathic tone (Beissner et al., 2013). The meta-analysis performed by Beissner and colleagues investigated noninvasive functional neuroimaging studies during waking state. However, we found that neural activity in the DMN is only correlated with parasympathetic modulation in N1, and not in the deeper sleep stages. This raises the question whether the regulation of the DMN of parasympathetic tone is mainly a property of waking and light sleep, and provides further evidence that the autonomic network during waking is most likely different from sleep. The organization of the autonomic network is also a function of sleep stage, as can be seen in Figure 3. Furthermore, similar to a previous finding (Kim et al., 2019), where it is shown that the correlation between neural firing rate and the cardiac cycle length could be positive or negative for different units in medial structures of humans during waking state. We found both positive and negative correlation coefficients for different channel pairs in these same structures during sleep (Fig. 3*A–C*), Within the same region, we found sites where the high γ activity is completely uncorrelated with HRV while simultaneously finding sites exhibiting a strong correlation. This suggests a spatial organization of the autonomic network that is at a finer scale than the ROIs investigated in this work. Finally, the interpatient mean correlation coefficients of high γ band and autonomic modulation are stable after mediating for heart rate and mildly affected when mediating for δ and θ band activity, providing further evidence that our results are specific to HG and its relationship to autonomic tone.

### Role of hippocampus

The hippocampus has been shown to modulate sympathetic contributions in the central autonomic network (CAN) during noninvasive functional neuroimaging waking studies (Beissner et al., 2013). However, little is known about the role of the hippocampus in autonomic modulation in sleep. We have shown that, during sleep, the HG in the anterior hippocampus has a stronger correlation with parasympathetic modulation as compared with the posterior hippocampus. This could be because the anterior hippocampus has stronger connections with areas of the brain that is known to modulate the autonomic system as compared with the posterior hippocampus, with direct connections found in anterior hippocampus with amygdalar nuclei (Ranganath and Ritchey, 2012; Poppenk et al., 2013; Strange et al., 2014). Most interestingly, this overall effect in the anterior hippocampus is significantly reduced when conditioning the correlation of high γ and autonomic tone on δ band activity in all sleep stages (Fig. 4). The posterior hippocampus’s overall effect is less affected when conditioning on δ band activity. The percentage of statistically correlated channels is significantly reduced in both the anterior and posterior hippocampus, as shown in Figure 5. Previous studies have shown that δ band modulates activity in high γ band during sleep (Csercsa et al., 2010; Halgren et al., 2018), and we have shown in this work that this modulation affects both hippocampal-ANS average correlations and the percentage of correlated channels. Further analysis is needed to understand the directionality and time scale of the hippocampal-ANS connection during sleep.

### Cortical interactions

The CAN in humans has been shown to consist of several cortical regions such as the anterior cingulate cortex (ACC), ventromedial prefrontal cortex, amygdala, and insula during waking studies (Beissner et al., 2013). The insula, in particular, has been studied extensively in its involvement in autonomic arousal (Critchley et al., 2000; Harper et al., 2000; Critchley, 2005; Rolls, 2016) and is also correlated with interoceptive awareness (Critchley et al., 2004). Although Figure 3*D–F* did not show a statistically significant overall trend in the correlation between insula high γ activation and autonomic modulation, we did observe that the percentage of correlated channels in the insula, most notably in N3, is substantially higher than all other regions (with five channel pairs collected across four patients), and is not heavily suppressed by removing the effect of δ band activity. This supports the claim that the insula is a major hub in the CAN, especially during deep sleep. Figure 3*A–C* shows that within insula (like other areas) bipolar channel pairs could have either sympathetic and parasympathetic coupling with high γ activity, providing evidence that the insula does not have a monolithic relationship with autonomic regulation, but there could exist a finer spatial organization that contributed to either sympathetic or parasympathetic modulation. Clearly, further work is required to understand how different areas within each subregion of the cortex are related to autonomic tone.

Many other cortical sites have been established to modulate autonomic tone, such as the orbitofrontal cortex (Kringelbach, 2005), lateral temporal lobe (Beissner et al., 2013), and prefrontal lobe (Thayer et al., 2012). We have shown that these areas are heavily involved in autonomic modulation during sleep and that their effect is affected by the sleep stage. We have also shown that both the lateral parietal and medial occipito-parietal lobes could be considered part of the CAN during sleep, as they have an overall effect observed in N1 and N3, respectively. The CAN has also been established to include the cingulate cortex (Critchley et al., 2003, 2005), yet we did not observe an overall statistically significant correlation in any sleep stage, whether mediating for δ band activity or not (Figs. 3, 4). Figure 5 shows that the percentage of correlated channels between cingulate cortex high γ activity and autonomic tone is more pronounced after removing the effect of the δ band, yet overall, the percentage of correlated channels is low in comparison to other anatomic regions (∼12% when removing δ band effect).

### Future work

We hope that this paper serves as a starting point for future researchers to understand CNS-ANS interaction during sleep. Since our method has low temporal resolution (in the order of minutes), as well as limited cortical coverage, it is difficult to infer directionality. Therefore, further analysis is needed to understand the dynamics of the interaction between cortical or hippocampal structures and the ANS by implementing measures of autonomic modulations that operate in the order of seconds or hundreds of milliseconds, such as a transient increase in heart rate, as well as using other measures such as galvanic skin response. Having a higher temporal resolution will enhance our understanding of what processes are generating the average correlations and connectivity between neural activity and HRV, as shown in Figure 6. In this study, we focused our analyses mainly on sleep periods, because of the lack of any potentially confounding behavioral or cognitive activity that could influence autonomic tone, such as moving or being in an anxious state. It would be important to extend these findings to the waking state using long-term intracranial recordings. This would lead to a direct comparison to noninvasive functional neuroimaging studies that leveraged either short-term resting state analyses or a trial-based experimental approach. Additionally, a thorough investigation of the interplay between frequency bands along with the background arrhythmic 1/f noise and their correlations with autonomic tone is needed to paint a fuller picture of the relationship between the CNS and ANS. Further studies investigating the CNS-ANS axis during waking state using intracranial electrophysiological recordings is also needed to compare with previous work that primarily used noninvasive recording modalities such as fMRI and PET.

Through understanding the interaction between the brain and the autonomic state, therapeutic modalities such as deep brain stimulation could be enhanced. Autonomic conditions that could be potentially managed through DBS include hypertension, asthma, and obstructive sleep apnea (Hyam et al., 2012). Better understanding of CAN would facilitate the modulation of blood pressure, respiration, and heart rate by stimulating areas in the brain that are part of the CAN. Multiple sleep pathologies such as sleep apnea, insomnia, and sudden death syndrome in epilepsy are related to abnormalities in autonomic regulation (Tobaldini et al., 2013). More specifically, primary insomnia patients exhibit changes in the correlation between HRV and δ band activity during sleep (Tobaldini et al., 2013). Vagal tone is also abnormal during slow-wave sleep in patients with refractory epilepsy (Jansen et al., 2011). This work could provide a gateway to understanding the relationships between the CNS-ANS in different pathologies and their relationship to sleep stage.

## Synthesis

Reviewing Editor: Michaël Zugaro, CNRS, Collège de France, Inserm Decisions are customarily a result of the Reviewing Editor and the peer reviewers coming together and discussing their recommendations until a consensus is reached. When revisions are invited, a fact-based synthesis statement explaining their decision and outlining what is needed to prepare a revision will be listed below. The following reviewer(s) agreed to reveal their identity: Enzo Tagliazucchi. Note: If this manuscript was transferred from JNeurosci and a decision was made to accept the manuscript without peer review, a brief statement to this effect will instead be what is listed below.

The manuscript investigates the relationship between autonomic tone and cortical and hippocampal activity in humans during sleep. By using high gamma (HG) power as a proxy for synchronous firing in neuronal populations, a correlation is described between heart rate variability (HRV) and endogenous brain activity, in particular in the lateral temporal lobe during non-rapid eye movement stage 3 (N3) sleep. This contrasts with previous reports using fMRI or PET in awake subjects, which is interpreted as evidence for modulation by state-dependent factors.

This study was previously evaluated, but not retained for publication, at J.Neurosci. then forwarded to eNeuro. It has now been evaluated by two reviewers, including one involved in the previous evaluation. This reviewer found that the clarifications concerning the low inter-parient correlation values (compared to relatively high values for single participants), combined with an extensive discussion of the potential sources of this variability, have contributed to dissipate previously expressed concerns.

Both reviewers agree that the topic is of high interest and that the study could provide novel and timely insights into central-autonomic interactions in humans. Nonetheless, issues have been raised regarding the methodological approach as well as the statistical presentation, which currently impede the evaluation of the results and conclusions.

Below is a list of points raised in the review process.

Major

The main reservations concern some of the authors' choices in computing and reporting statistics.

ll. 220-222: If I understand the Methods section correctly, the authors used FDR to correct for multiple comparisons within each ROI (across electrode pairs), but not across ROIs. I would be interested to know why the authors did not apply MC correction across ROIs as well.

ll. 226-227: The authors report that they first pooled and then averaged ROI-specific correlation coefficients for a subsequent t-test. I suspect (and hope) that the authors did not compute the mean of raw correlation coefficients (which would be improper), but first used an inverse tangent function to compute the mean Fisher z-transformed correlations. However, I could not find a reference to any transformation prior to computing mean correlations or subjecting them to statistical testing (ll. 229-230 and multiple instances later). Please clarify.

ll. 238-239: I fundamentally disagree with the authors' definition of coupling as some percentage of significantly correlated pairs. There is a well-established concept of central-peripheral coupling - namely, phase-amplitude coupling - which the authors cite themselves later (l. 402). Therefore, I wonder why the authors chose not to employ an established coupling measure (such as the modulation index, for example), but instead relied on an in-house definition of the very same concept. I would argue that the coupling terminology (as defined by the authors) is somewhat misleading or at least prone to misinterpretations.

ll. 269-271: I could not find any information as to how assumptions of the ANOVA were tested and what the results of these tests were. Again, it appears as if the authors simply entered the raw correlation coefficients into the ANOVA. Given the underlying data, I am sceptical whether a parametric test is indeed the correct choice, but I would be happy to be convinced otherwise by transparent statistics.

ll. 273-274: The model comparison described here is incorrect. In order to compare the fit of two models, the simpler model must be fully nested within the more complex model. I recognise that the authors later describe exactly that (ll. 358-360), but the discrepancy between both instances makes it impossible to judge the result of the LMEM comparison. This is complicated further by the authors' incomplete reporting of statistics (see separate point below): What is the result of the theoretical likelihood test, including test statistic, degrees of freedom, and interpretation of the chi squared statistic? What is the respective fit of each model (AIC, BIC, ...)? What are the significant effects shown within each model? The authors claim that LMEM provides 'strong evidence', which the reader cannot possibly evaluate without proper reporting of the underlying analyses.

ll. 302-303: The authors claim there is a shift towards parasympathetic balance, depending on sleep stage. How were the HFnorm differences tested within each participant? What is the statistical test, and what are the results? What was the authors' reasoning against conducting a group-level statistic?

ll. 305-308: The same is true here. How were differences of average HFnorm against healthy individuals tested? Did the authors actually conduct a test against the averages provided by Busek et al. (2005)? If so, what are the results? Furthermore, how were the HG differences compared across sleep stages? Once again, the authors unfortunately report neither the test nor its results.

ll. 395-397: How was the decrease from n = 14 to n = 10 significant locations assessed statistically? If this difference is to be interpreted, it should be validated by a statistical test.

ll. 429-432: The same is true here. How did the authors determine whether the percentages did indeed drop significantly?

As a general major concern, I would like to point out that the overall reporting of statistical results is insufficient. Just to briefly name some examples, correlation coefficients require degrees of freedom (e.g. l. 331), the p value cannot be reported as equal to zero (l. 332 and multiple other instances), and test statistics from binomial tests (l. 334), all t-tests, and the likelihood ratio test (l. 359-360) are missing. F-statistics from the ANOVA (e.g. l. 349) require the appropriate degrees of freedom (numerator and denominator of the F ratio). I would urge the authors to revise both their Methods and Results sections with a focus on a transparent reporting of their approach and the results it yielded.

Minor

l. 5: Given the somewhat ubiquitous definitions of frequency bands across the literature, I would appreciate an early definition of high gamma already in the Abstract.

l. 11: The mentioning of delta band activity (again, please specify the frequency range) comes a bit out of the blue and definitely requires some introduction.

l. 14: Should read 'such as'.

ll. 18-19: The Abstract closes with a summary of findings already reported above. The authors could significantly increase the perceived impact of their manuscript by outlining potential interpretations or starting points for interesting future work.

ll. 33-45: The Introduction includes surprisingly few citations, particularly within the first two paragraphs. As some of the claims and reports of prior work are quite substantial, I suggest adding at least some references.

l. 59: Why is the insula singled out here? Again, there is no reference and no prior introduction that the insula could be of interest; I suggest appropriately revising this paragraph.

l. 86: For quite some time, I was puzzled by the actual number of patients, as the Abstract mentions N = 16. The issue is only resolved in parentheses much later (ll. 169-170), which is unfortunate. I suggest including some information about the to-be-rejected patient already in the Patient Selection section and to describe age and sex distribution of the final sample, not the original one.

ll. 133-134: Were any channels rejected during visual inspection? If so, how many?

l. 135: Again, the delta band is mentioned without any previous reasoning or introduction. The manuscript would greatly profit from an explanation why delta is important and why potential interactions with high gamma should be considered.

l. 164: 'We fitted a line' is overly vague. What kind of line was fitted, probably some kind of least squares? Please specify.

l. 225: In this and other instances, the t-test is referred to as 'student t-test test', which is highly irregular.

Figure 2: In contrast to the other figures, the labels and legends of Fig. 2 are barely readable. The letters A, B, and C are unnecessarily enlarged. While I appreciate the demonstration shown in panel B, I honestly do not know what to gain from panel C. Is this to be just an example, computed from the short cut-out shown in panel B?

## Author Response

Synthesis of Reviews:

Synthesis Statement for Author (Required):

The manuscript investigates the relationship between autonomic tone and cortical and hippocampal activity in humans during sleep. By using high gamma (HG) power as a proxy for synchronous firing in neuronal populations, a correlation is described between heart rate variability (HRV) and endogenous brain activity, in particular in the lateral temporal lobe during non-rapid eye movement stage 3 (N3) sleep. This contrasts with previous reports using fMRI or PET in awake subjects, which is interpreted as evidence for modulation by state-dependent factors.

This study was previously evaluated, but not retained for publication, at J.Neurosci. then forwarded to eNeuro. It has now been evaluated by two reviewers, including one involved in the previous evaluation. This reviewer found that the clarifications concerning the low inter-parient correlation values (compared to relatively high values for single participants), combined with an extensive discussion of the potential sources of this variability, have contributed to dissipate previously expressed concerns.

Both reviewers agree that the topic is of high interest and that the study could provide novel and timely insights into central-autonomic interactions in humans. Nonetheless, issues have been raised regarding the methodological approach as well as the statistical presentation, which currently impede the evaluation of the results and conclusions.

Below is a list of points raised in the review process.

Major

The main reservations concern some of the authors' choices in computing and reporting statistics.

ll. 220-222: If I understand the Methods section correctly, the authors used FDR to correct for multiple comparisons within each ROI (across electrode pairs), but not across ROIs. I would be interested to know why the authors did not apply MC correction across ROIs as well.

To determine which electrode pairs are labeled as statistically significant (looking at the individual electrode pair level), we used the FDR correction in each ROI separately. Our reasoning being is that there is an uneven distribution of electrode pairs in each ROI. We have the least number of electrode pairs in the insula, as opposed to hippocampal locations. If we applied an FDR correction across all ROI's, then the results can be biased towards the ROI's with the most number of electrode pairs. This result was intended to give the reader intuition that within each ROI, we can find electrode pairs that are statistically correlated with autonomic tone.

To determine, on a macro level, which ROI's have an average correlation coefficient across patients that statistically differ from zero, we applied an FDR correction across ROI's. The percent of correlated channels in NREM stages 1, 2, and 3 are 19.6%, 51.5%, and 46.4%. These percentages are almost identical to the percentages we reported in line 314 using ROI specific MC correction. We added an extra sentence to include these results in the 'Overall correlation effect present in multiple sites and is dependent on NREM sleep stage' subsection.

ll. 226-227: The authors report that they first pooled and then averaged ROI-specific correlation coefficients for a subsequent t-test. I suspect (and hope) that the authors did not compute the mean of raw correlation coefficients (which would be improper), but first used an inverse tangent function to compute the mean Fisher z-transformed correlations. However, I could not find a reference to any transformation prior to computing mean correlations or subjecting them to statistical testing (ll. 229-230 and multiple instances later). Please clarify.

Thank you for pointing this out. We have considered applying the Fisher z-transformation on the raw correlation scores while writing this paper. However, looking at the distribution of correlation coefficients, the results would barely be changed because the Fisher z-transformation doesn't significantly alter values within the range of correlation in our results (from -0.55 to 0.54). However, to adhere to statistical rigor, we have applied the Fisher z-transformation on the correlation coefficients analyzed in this work and updated all the values and statistical tests accordingly. We replaced 'mean r' to 'mean z' to indicate that we are applying statistical analyses on the z-scores, not the raw correlation coefficients. Figures 3 and 4 have also been updated using the z-scores. Aside from small deviations in the values, none of our results and conclusions have changed except that the number of statistically correlated ROI's across all sleep stages when using theta-band as a mediating variable is 9 instead of 10.

ll. 238-239: I fundamentally disagree with the authors' definition of coupling as some percentage of significantly correlated pairs. There is a well-established concept of central-peripheral coupling - namely, phase-amplitude coupling - which the authors cite themselves later (l. 402). Therefore, I wonder why the authors chose not to employ an established coupling measure (such as the modulation index, for example), but instead relied on an in-house definition of the very same concept. I would argue that the coupling terminology (as defined by the authors) is somewhat misleading or at least prone to misinterpretations.

We understand the reviewer's confusion regarding the definition of coupling. We used the term 'coupling' in our paper because, at the time, we believed it most adequately captured the concept we were trying to convey. However, future readers might be mislead, as the reviewer pointed out, and we think it would be best to change the terminology in the paper to 'percent of correlated channels' rather than 'coupling'. We have also used the term 'undirected connectivity in parts of the text as a quick reference to the analysis. However, we mainly use 'percentage of correlated channels' in the text to avoid confusion. This would be more straightforward.

Looking at the literature, we could not find a metric that is exactly what we have done in our analysis. We do not see how the modulation index is equivalent to our in-house 'coupling' analysis. To our knowledge, the modulation index is the extent of modulation done on the carrier signal, which is different than what we are trying to investigate. Additionally, the well-known metric that best describes what we have done is Undirected Network Density (UND). UND looks at the ratio of connections to all possible connections. However, since in our work we do not investigate the connection between different brain regions, this might cause some confusion especially if the reader is well versed in brain network analysis.

ll. 269-271: I could not find any information as to how assumptions of the ANOVA were tested and what the results of these tests were. Again, it appears as if the authors simply entered the raw correlation coefficients into the ANOVA. Given the underlying data, I am sceptical whether a parametric test is indeed the correct choice, but I would be happy to be convinced otherwise by transparent statistics.

Thank you for pointing this out. We have tested the Gaussianity of the residuals generated from the fitted ANOVA model using the Kolmogorov-Smirnov test and found that we cannot reject the null hypothesis that the residuals come from a normal distribution. We will include this finding in the text as well.

ll. 273-274: The model comparison described here is incorrect. In order to compare the fit of two models, the simpler model must be fully nested within the more complex model. I recognise that the authors later describe exactly that (ll. 358-360), but the discrepancy between both instances makes it impossible to judge the result of the LMEM comparison. This is complicated further by the authors' incomplete reporting of statistics (see separate point below): What is the result of the theoretical likelihood test, including test statistic, degrees of freedom, and interpretation of the chi squared statistic? What is the respective fit of each model (AIC, BIC, ...)? What are the significant effects shown within each model? The authors claim that LMEM provides 'strong evidence', which the reader cannot possibly evaluate without proper reporting of the underlying analyses.

Thank you for pointing this discrepancy out. There is indeed a mistake in our reporting in lines 273-274. We applied the methodology described in lines 358-360 and have edited the text accordingly.

Additionally, we understand the reviewer's concern regarding the lack of transparency in our statistical analysis. We would like to mention that firstly the statistical test was done using the 'fitlme' and 'compare' functions in MATLAB. These functions test whether the more complex LMEM is a better fit than the simpler LMEM using the nesting method. Our reasoning for not diving deeper into the statistics of the LMEMs is that we used the LMEM analysis as a control to determine whether the patient-specific variation is driving our results derived in our main analyses, but not for predicting correlation coefficients. However, we have now have edited the text to include the result of the theoretical likelihood test (test statistic, and degrees of freedom of the chi-squared distribution), (χ^2 (38)=184.5, p = 3.91 ×10^(-21)). The AIC for the simple and more complex models are -986 and -1094, respectively. The BIC of the simple and more complex models are -971 and -893 respectively. The decrease in the BIC in the more complex model could be due to the large sample size n=1008, and the large difference in the degrees of freedom, 38, which in turn would be punishing the more complex model.

The intention here is to show that there is information beyond patient-specific variations and to support our main observations and analyses. The result of our LMEM analysis is meant to be looked at alongside the N-way ANOVA to show that sleep stage and the anatomical location, alongside their interactions, provide information on the correlation between autonomic tone and high gamma-band activity. We do not use the LMEM to be able to predict the correlation coefficients, but rather as a control to see if patient-specific variations (regardless of anatomical location and sleep stage) are the main drivers of our results.

ll. 302-303: The authors claim there is a shift towards parasympathetic balance, depending on sleep stage. How were the HFnorm differences tested within each participant? What is the statistical test, and what are the results? What was the authors' reasoning against conducting a group-level statistic?

Thank you for making this point. We admit that in this part of the paper, we did not apply statistical rigor when we made these observations. We intended to provide some qualitative description of the data to give the reader an idea of how the heart rate variability is behaving according to sleep stage. We made this assessment by applying an ANOVA test on each subject with heart rate variability as the response variable and sleep stage as the grouping variable. We now realize that we were actually reporting the number of subjects were the HFnorm mean in each sleep stage are statistically different. Our statement that the HFnorm in N3 is greater than N1 was made visually.

After revisiting our analysis, we concluded that it would be best to apply a group level statistical analysis rather than a patient-specific analysis to summarize and simplify the results. We did this by subtracting the patient-specific mean from the HFnorm to focus on the relative change in HFnorm that is sleep stage dependent. Using the baseline HFnorm as a response variable and sleep stage as a grouping variable, we applied a one-way ANOVA to find that sleep stage affects baseline HFnorm across subjects F(2,42)=11.28, p=0.0001. We then applied a post-hoc analysis using Tukey's range test and found that baseline HFnorm in N3 is greater than N1 and N2 (p=0.0001 and p=0.0452 respectively), indicating a shift towards a parasympathetic state when entering deep sleep. We tested the Gaussianity of the residuals generated from the ANOVA test using the Kilmorgnov-Smirnov test and could not reject the hypothesis that the residuals come from a normal distribution.

ll. 305-308: The same is true here. How were differences of average HFnorm against healthy individuals tested? Did the authors actually conduct a test against the averages provided by Busek et al. (2005)? If so, what are the results? Furthermore, how were the HG differences compared across sleep stages? Once again, the authors unfortunately report neither the test nor its results.

When making this claim, we did not apply any statistical tests, but rather wanted to show that the means of the HFnorm in the patients we analyzed are around the same values for healthy subjects. In Busek et al (2005), the HFnorm found in Stage 2 and Stage 4 non-REM sleep were 0.58 and 0.6 respectively. We could not find any research article that specifically looked at the HFnorm for N1, N2, and N3. We think the best approach is to change the text in the paper to say that the HFnorm we obtained in our work is similar to previous work looking at healthy patients. The intention with this statement is not to make concrete statistical claims regarding if the HFnorm values derived in this work matches healthy subjects, but rather give the reader some intuition about our data and reassurance that the heart rate variability metrics generated are reasonable. We changed the text (lines 310-312) to “These values were similar to previous work analyzing the HFnorm in healthy individuals (Busek et al., 2005).”

In regards to our claim regarding high gamma band activity, again, we made it by visually examining the data without any statistical tests. The intention was to give the reader intuition about our data and how high gamma band activity is related to sleep stage. We do realize that our claim needs to be backed up by statistical analysis. Firstly, we normalized each patient's high gamma band activity according to their baseline to remove patient specific shifts of high gamma band activity and to only investigate the relative changes that are sleep stage dependent. We then applied a one-way ANOVA test using high gamma band activity patient-specific baseline as the response variable and sleep stage as the grouping variable. High gamma activity is dependent of sleep stage F(2,42)=14.48, p=0.000065. We then used the multcompare function in MATLAB to apply posthoc analysis in each subject using Tukey's test and found that baseline high gamma activity in N3 is lower than N1 and N2 (p=1.172 x10^-5 and p=0.0036 respectively). We tested the Gaussianity of the residuals generated from the ANOVA test using the Kilmorgnov-Smirnov test and could not reject the hypothesis that the residuals come from a normal distribution.

ll. 395-397: How was the decrease from n = 14 to n = 10 significant locations assessed statistically? If this difference is to be interpreted, it should be validated by a statistical test.

We realize the reviewer's concern regarding this point. However, our paper's conclusions and results do not rely on this observation. We intended to make a simple qualitative observation after analyzing all of our data. The text describing this observation might be too forward and requires statistical rigor. We simply have two data points, n=14 and n=10, so do we do not see what kind of statistical test is appropriate.

To alleviate this issue, we believe the best step is to soften the claims in the text and avoid making concrete conclusions from this observation. We will change the text in the paper to something resembling “We conclude that the overall correlations in the cortex and hippocampus of high gamma activity and autonomic tone is generally maintained after mediating for delta band but there might exist a small effect.” (lines 429-430)

ll. 429-432: The same is true here. How did the authors determine whether the percentages did indeed drop significantly?

We previously applied pairwise Wilcoxon signed rank tests to make our conclusions and internally debated whether it would be necessary to include the statistics. We will make the appropriate adjustments in the text to include our statistical results. Please see lines 465-470)

As a general major concern, I would like to point out that the overall reporting of statistical results is insufficient. Just to briefly name some examples, correlation coefficients require degrees of freedom (e.g. l. 331), the p value cannot be reported as equal to zero (l. 332 and multiple other instances), and test statistics from binomial tests (l. 334), all t-tests, and the likelihood ratio test (l. 359-360) are missing. F-statistics from the ANOVA (e.g. l. 349) require the appropriate degrees of freedom (numerator and denominator of the F ratio). I would urge the authors to revise both their Methods and Results sections with a focus on a transparent reporting of their approach and the results it yielded.

Thank you for pointing this out. We will make the necessary adjustments in the text and try to be as transparent as possible with the statistics.

In regards to the correlation coefficients degrees of freedom, we mainly report the mean correlation coefficient across patients, therefore, there is no degree of freedom to report. We do not report the correlation coefficient of any individual bipolar channel as we are mainly interested in the patient-wide trends. For the binomial test, we used the binomial formula to calculate the p-value directly, so we are unsure what test statistic you are referring to. Finally, we have reported all the p-values as their exact number (not equals to zero or using an inequality). This is due to the guidelines described in the manuscript preparation guidelines. If you see that some of the very small p-values should be changed to an inequality, please go ahead and change them or we can do it ourselves.

Minor

l. 5: Given the somewhat ubiquitous definitions of frequency bands across the literature, I would appreciate an early definition of high gamma already in the Abstract.

We have made the clarification in the abstract.

l. 11: The mentioning of delta band activity (again, please specify the frequency range) comes a bit out of the blue and definitely requires some introduction.

We have made the necessary clarifications.

l. 14: Should read 'such as'.

We have edited this.

ll. 18-19: The Abstract closes with a summary of findings already reported above. The authors could significantly increase the perceived impact of their manuscript by outlining potential interpretations or starting points for interesting future work.

We edited the abstract to address this concern.

ll. 33-45: The Introduction includes surprisingly few citations, particularly within the first two paragraphs. As some of the claims and reports of prior work are quite substantial, I suggest adding at least some references.

We have added additional citations in the introduction to address this concern

l. 59: Why is the insula singled out here? Again, there is no reference and no prior introduction that the insula could be of interest; I suggest appropriately revising this paragraph.

We agree that our mentioning of Insula came out of the blue. We have ommited mentioning Insula in this paragraph to avoid the abrupt confusion.

l. 86: For quite some time, I was puzzled by the actual number of patients, as the Abstract mentions N = 16. The issue is only resolved in parentheses much later (ll. 169-170), which is unfortunate. I suggest including some information about the to-be-rejected patient already in the Patient Selection section and to describe age and sex distribution of the final sample, not the original one.

Thank you for pointing this out. We have made the necessary edits to avoid the confusion regarding the number of patients.

ll. 133-134: Were any channels rejected during visual inspection? If so, how many?

32 out of 368 bipolar pairs were visually labeled as bad. We updated Table 1 to remove channels that were visually rejected and updated the text in the 'summary of data' subsection.

l. 135: Again, the delta band is mentioned without any previous reasoning or introduction. The manuscript would greatly profit from an explanation why delta is important and why potential interactions with high gamma should be considered.

We have mentioned in line 82 that delta band upstates and downstates are coupled with high gamma band activity during sleep.

l. 164: 'We fitted a line' is overly vague. What kind of line was fitted, probably some kind of least squares? Please specify.

Yes, we used a linear regression model to fit the line. We have made the necessary edits in the text.

l. 225: In this and other instances, the t-test is referred to as 'student t-test test', which is highly irregular.

This was a typo. We have made the appropriate edits.

Figure 2: In contrast to the other figures, the labels and legends of Fig. 2 are barely readable. The letters A, B, and C are unnecessarily enlarged. While I appreciate the demonstration shown in panel B, I honestly do not know what to gain from panel C. Is this to be just an example, computed from the short cut-out shown in panel B?

We have enlarged the labels and legends to become more readable and reduced the sizes of A,B and C. Panel C is used as an example to demonstrate the difference in HFnorm for the two intervals displayed in figure 2B. We used red and blue to match the graphs of the spectral estimations for the sympathetic and parasympathetic RR-interval epochs/
