# Supplementary material for: High γ Activity in Cortex and Hippocampus Is Correlated with Autonomic Tone during Sleep
Source: eNeuro. 2021 Nov 17;8(6):ENEURO.0194-21.2021. doi: 10.1523/ENEURO.0194-21.2021 (PMC8607912; doi:10.1523/ENEURO.0194-21.2021)
Supplement: Extended Data Table 5-1 — The percentage of correlated channels (bootstrapped) using the mean correlation and partial correlation in different sleep stages for each ROI. Download Table 5-1, DOCX file. [file enu-eN-NWR-0194-21-s02.docx]

***Extended Table 5-1: The percentage of correlated channels (bootstrapped) using the mean correlation and partial correlation in different sleep stages for each ROI.***

|  | Mean % of statistically correlated channel pairs. | | | Mean % of statistically partially correlated channel pairs. | | |
| --- | --- | --- | --- | --- | --- | --- |
| Location | N1 | N2 | N3 | N1 | N2 | N3 |
| Anterior Hippocampus | 7.8 | 5.9 | 22.3 | 0.6 | 1.2 | 2.5 |
| Posterior Hippocampus | 2.2 | 2.8 | 14.1 | 1.6 | 2.2 | 4.8 |
| Cingulate | 0.6 | 3.0 | 8.7 | 0.6 | 2.0 | 12.8 |
| Insula | 6.3 | 12.9 | 53.1 | 8.4 | 11.1 | 40.1 |
| Lateral Temporal | 9.1 | 3.5 | 17.0 | 6.4 | 5.5 | 15.1 |
| Lateral Occipital | 1.4 | 4.1 | 21.4 | 1.6 | 2.8 | 19.0 |
| Lateral Parietal | 2.1 | 4.4 | 5.5 | 1.9 | 4.2 | 9.8 |
| Medial Occipito-Parietal | 1.5 | 3.2 | 5.2 | 1.5 | 2.4 | 5.5 |
| Medial Temporo-occipital | 2.5 | 1.9 | 1.0 | 1.6 | 1.1 | 0.9 |
| Orbitofrontal | 2.5 | 3.5 | 20.4 | 1.7 | 2.5 | 5.1 |
| Paracentral | 4.4 | 7.0 | 4.8 | 3.4 | 5.1 | 5.2 |
| Prefrontal | 0.8 | 4.1 | 3.3 | 0.5 | 2.6 | 3.8 |
